# Impact of biological aging on arterial aging in American Indians: findings from the Strong Heart Family Study

**DOI:** 10.18632/aging.101013

**Published:** 2016-08-11

**Authors:** Hao Peng, Yun Zhu, Fawn Yeh, Shelley A. Cole, Lyle G. Best, Jue Lin, Elizabeth Blackburn, Richard B. Devereux, Mary J. Roman, Elisa T. Lee, Barbara V. Howard, Jinying Zhao

**Affiliations:** ^1^ Department of Epidemiology, Tulane University School of Public Health, New Orleans, LA 70112, USA; ^2^ Center for American Indian Health Research, University of Oklahoma Health Science Center, Oklahoma City, OK 73104, USA; ^3^ Department of Genetics, Texas Biomedical Research Institute, San Antonio, TX 78227, USA; ^4^ Missouri Breaks Industries Research Inc, Timber Lake, SD 57656, USA; ^5^ Department of Biochemistry and Biophysics at the University of California, San Francisco, CA 94143, USA; ^6^ Greenberg Division of Cardiology, Weill Cornell Medical College, New York, NY 10065, USA; ^7^ MedStar Health Research Institute, Hyattsville, MD 20782, USA

**Keywords:** American Indians, arterial aging, arterial stiffness, biological aging, leukocyte telomere length, Strong Heart Family Study

## Abstract

Telomere length, a marker of biological aging, has been associated with cardiovascular disease (CVD). Increased arterial stiffness, an indicator of arterial aging, predicts adverse CVD outcomes. However, the relationship between telomere length and arterial stiffness is less well studied. Here we examined the cross-sectional association between leukocyte telomere length (LTL) and arterial stiffness in 2,165 American Indians in the Strong Heart Family Study (SHFS). LTL was measured by qPCR. Arterial stiffness was assessed by stiffness index β. The association between LTL and arterial stiffness was assessed by generalized estimating equation model, adjusting for sociodemographics (age, sex, education level), study site, metabolic factors (fasting glucose, lipids, systolic blood pressure, and kidney function), lifestyle (BMI, smoking, drinking, and physical activity), and prevalent CVD. Results showed that longer LTL was significantly associated with a decreased arterial stiffness (β=-0.070, *P*=0.007). This association did not attenuate after further adjustment for hsCRP (β=-0.071, *P*=0.005) or excluding participants with overt CVD (β=-0.068, *P*=0.012), diabetes (β=-0.070, *P*=0.005), or chronic kidney disease (β=-0.090, *P*=0.001). In summary, shorter LTL was significantly associated with an increased arterial stiffness, independent of known risk factors. This finding may shed light on the potential role of biological aging in arterial aging in American Indians.

## INTRODUCTION

Telomeres are repetitive DNA sequences and their associated proteins at the end of chromosomes. They protect against chromosomal degradation and prevent chromosomes from fusion, thus play a critical role in maintaining genomic stability [1]. Telomere length shortens during each round of cell division, and when it reaches a critical length, cells stop division and enter senescence [2]. Thus, telomere length has been considered a marker of biological aging. Shorter telomere length has been associated with an increased risk of various age-related diseases including cardiovascular disease (CVD) [3] and its risk factors, e.g., obesity [4], hypertension [5], diabetes [6, 7], and dyslipidemia [8]. In a large cohort of American Indians, we have recently demonstrated prospective associations of leukocyte telomere length (LTL) with diabetes [6] and carotid atherosclerosis [9] as well as cross-sectional association of LTL with obesity [4]. All these conditions have been related to arterial aging characterized by increased stiffness in the arteries [10].

Arterial stiffness refers to the reduced elasticity of an artery in response to pressure changes. It increases with age [11] and predicts adverse CVD outcomes [12, 13], thus representing a key feature of arterial or vascular aging [14]. Shorter telomere length has been found in vascular smooth muscle cells from aging artery [15]. These findings indicate a probable association between telomere length and arterial stiffness, however, this relationship has been less well studied in human populations [16-21]. Among the very few existing studies examining the relationship between telomere length and arterial stiffness in human populations, sample sizes were in general small, and the investigations were restricted to European populations [18-20]. Moreover, most previous studies failed to extensively control for known coronary risk factors. The goal of this study is to examine whether LTL is associat ed with arterial stiffness in a large cohort of American Indians participating in the Strong Heart Family Study (SHFS), independent of established coronary risk factors.

## RESULTS

### Baseline characteristics

The current analysis included a total of 2,165 American Indian participants (883 men, 1,282 women) with a mean age of 41 ± 17 years (aged 14 to 93). The median stiffness index β was 3.24 (interquartile range: 2.36-4.58). Prevalent CVD, diabetes, and chronic kidney disease accounted for 5%, 18%, and 6% of the study population, respectively. Table 1 shows the baseline characteristics of study participants according to LTL quartiles. Compared to participants with longer LTL, those with shorter LTL had significantly higher body mass index, waist circumference, systolic blood pressure, and fasting glucose after controlling for age and sex (all *P <* 0.05). No significant difference was detected in other listed covariates across the LTL quartiles.

**Table 1:** Characteristics of study participants according to LTL quartiles (n=2,165)

Characteristics	LTL quartiles (T/S ratio)				*P* for trend*
	Q1(≤0.84)	Q2(0.85 - 0.98)	Q3(0.99 - 1.13)	Q4(> 1.13)	
N	540	542	544	539	-
Education level, years	12.4±2.3	12.5±2.3	12.2±2.2	12.0±2.2	0.503
Physical activity, steps/day	5138±3787	5469±3772	6141±3902	6605±4102	0.055
Body mass index, kg/m^2^	31.7±6.5	31.6±7.6	31.0±6.9	29.3±7.2	<0.001
Waist circumference, cm	104.8±17.4	103.3±18.0	101.2±16.5	96.6±16.7	<0.001
Systolic blood pressure, mmHg	125.5±17.6	124.7±17.2	124.6±17.0	119.3±14.8	0.016
Diastolic blood pressure, mmHg	76.1±10.5	76.7±11.4	77.3±11.6	74.9±10.9	0.723
Fasting glucose, mg/dL	114.8±49.6	111.1±45.1	105.8±42.4	99.8±30.8	0.025
Total cholesterol, mg/dL	187.9±39.6	186.3±37.0	183.9±36.5	174.7±39.0	0.406
Triglycerides, mg/dL	186.3±269.3	176.7±143.2	166.0±148.6	149.0±191.9	0.354
LDL-C, mg/dL	101.4±31.7	101.3±30.3	101.4±31.3	94.1±27.8	0.690
HDL-C, mg/dL	52.1±14.9	51.7±14.7	50.9±15.0	51.8±14.5	0.944
eGFR, mL/min/1.73 m^2^	87.0±23.4	93.0±25.4	97.8±25.0	102.0±23.5	0.264

* Adjust for age and sex using GEE to account for family relatedness. LDL-C: low density lipoprotein cholesterol; HDL-C: high density lipoprotein cholesterol; eGFR: estimated glomerular filtration rate; LTL: leukocyte telomere length.

### Association between LTL and arterial stiffness

Table 2 shows the multivariate-adjusted association between LTL and log-transformed stiffness index β (log-SI). After adjusting for sociodemographics, study site, metabolic factors, lifestyle, and prevalent CVD, longer LTL was significantly associated with lower log-SI (β = −0.070, *P* = 0.007). Further adjusting for hsCRP did not attenuate the association between LTL and log-SI (β = −0.071, *P* = 0.005), indicating that the observed association between LTL and arterial stiffness may not be confounded by inflammation. This association remained significant after excluding participants with prevalent CVD (β = −0.068, *P* = 0.012), diabetes (β = −0.070, *P* = 0.005), or chronic kidney disease (β = −0.090, *P* = 0.001). These results indicated that the observed association between LTL and arterial stiffness may not be confounded by these chronic conditions.

**Table 2:** Multivariate-adjusted association between LTL and log-transformed stiffness index β in American Indians

Subgroups	No. of participants	Multivariate-adjusted*		Additionally adjusted for hsCRP†	
		β (SE) £	*P*-value	β (SE) £	*P*-value
All participants	2165	−0.070 (0.026)	0.007	−0.071 (0.025)	0.005
No CVD	2062	−0.068 (0.028)	0.014	−0.068 (0.027)	0.012
No diabetes	1778	−0.069 (0.026)	0.007	−0.070 (0.025)	0.005
No chronic kidney disease	2033	−0.089 (0.028)	0.001	−0.090 (0.027)	0.001

LTL: leukocyte telomere length; hsCRP: high-sensitivity C-reactive protein.

£ Decrease in log-transformed stiffness index β per unit increase in LTL (T/S ratio).

* Adjusting for sociodemographics (age, sex, education level), study site, metabolic factors (systolic blood pressure, fasting glucose, low- and high- density lipoprotein cholesterol, estimated glomerular filtration rate), lifestyle factors (body mass index, current smoking, current drinking, physical activity), and prevalent CVD.

† Further adjusting for high-sensitivity C-reactive protein.

### Results of sensitivity analysis

Additionally adjusting for medications on hypertension, diabetes, and hypercholesterolemia did not change the association between LTL and arterial stiffness (Supplementary Table S1), suggesting that the observed association between LTL and arterial stiffness may not be attributed to these medications.

## DISCUSSION

In a large population of American Indians, we found that leukocyte telomere length was significantly and inversely associated with arterial stiffness, independent of known risk factors. This finding provides initial evidence that biological aging measured by leukocyte telomere length may play a role in arterial aging in American Indians.

The inverse association between leukocyte telomere length and arterial aging observed in our study is in line with previous findings in experimental studies. For instance, arterial stiffness was significantly increased in *klotho*-deficient mice as compared to their age-matched wild-type littermates [22]. In addition, overexpression of the telomeric repeat binding factor 1 (*TERF1*) gene reduced telomere-associated DNA damage foci and pro-inflammatory cytokine expression in aging endothelial cells [23]. As *TERF1* gene encodes a protein that maintains telomere length,[24] this finding may suggest a protective effect of longer telomeres on the senescence of endothelial cells in the vascular system.

Several population studies have also reported an inverse association between leukocyte telomere length and arterial aging [16-20], though mixed results existed [21]. For instance, in a recent study including 303 Russians (mean age 52 years) free of CVD, arterial stiffness measured by carotid-femoral pulse wave velocity was negatively associated with LTL [18]. This association was confirmed in other ethnic groups including African-Americans [16] and European Caucasians [20]. Moreover, shorter telomere length was associated with other measures of vascular/arterial aging, e.g., carotid artery intimal medial thickness [25] and endothelial dysfunction [26]. Further, the inverse relationship between leukocyte telomere length and arterial aging was in agreement with our previous studies in American Indians demonstrating that shorter telomere length significantly predicted an increased risk of carotid atherosclerosis [9] and diabetes [6], both of which have been related to arterial stiffness [10]. While these results provided evidence for a role of biological aging in arterial stiffness, prior investigations were mostly of limited sample size and largely conducted in populations of European ancestry [18-20]. We are not aware of prior studies examining the association between LTL and arterial stiffness in American Indians. The current study included a large sample of American Indians, and our analysis extensively adjusted for known risk factors and chronic metabolic conditions. Our results suggest that accelerated biological aging may underlie the pathogenesis of arterial aging through pathways beyond known risk factors.

As medications on hypertension [27], diabetes [28], and hypercholesterolemia [29] may exert favorable effects on arterial aging, we thus performed additional analysis to examine whether the use of these medications may influence our results. It shows that additional adjustments for medications on hypertension, diabetes, and hyperlipidemia did not appreciably attenuate the association between LTL and arterial stiffness, indicating that the observed association may not be confounded by these medications. In addition, as previous studies reported that participants with overt CVD, diabetes, or chronic kidney disease had increased arterial stiffness [12, 13, 30, 31], we conducted separate analyses by excluding participants with prevalent CVD, diabetes, or chronic kidney disease. Results show that the association between telomere length and arterial stiffness persisted after excluding participants with these chronic conditions.

Inflammation and oxidative stress may underlie the observed association between telomere length and arterial aging. First, it is known that inflammation and oxidative stress are important contributors to arterial/vascular aging [32, 33]. Second, inflammation or oxidative stress has been shown to accelerate telomere shortening in both experimental [34, 35] and human studies [36, 37]. Thus, it is possible that the observed association between telomere length and arterial stiffness may be confounded by inflammation. To address this, we conducted additional analysis by further adjusting for hsCRP in the GEE model. It showed that the association between telomere length and arterial stiffness remained unchanged after further adjustment for hsCRP, indicating that the observed association may not be confounded by inflammation. Due to the lack of oxidative stress markers in the SHFS, we were unable to examine the influence of oxidative stress on our results. We are unaware of previous studies examining the relationship between oxidative stress, telomere length and arterial/vascular aging.

As far as we are aware, this is the first study to examine the association between telomere length and arterial stiffness in American Indians. Strengths of this study include the high quality telomere data, large sample size, and comprehensive adjustments for known risk factors, chronic metabolic conditions, as well as medications. However, several limitations should also be mentioned. First, the cross-sectional nature of our analysis precludes us from investigating the potential causal role of telomere shortening in arterial stiffness. Second, our findings were derived from American Indians whose cardiovascular profiles could be different from those with other ethnic backgrounds, and thus the generalizability of our results to other populations is unknown. However, our study population may serve as a model for other ethnic groups having high rates of diabetes and obesity. Third, leukocyte telomere length exhibits a wide range of inter-individual variation, and it also varies among cells in the same tissue and among chromosomes in the same cell [38]. In the present study, we measured telomere length in blood leukocytes but not arteries. However, previous studies have demonstrated that telomere length in different tissues may be highly correlated [39].

In summary, shorter leukocyte telomere length was significantly associated with an increased arterial stiffness in American Indians, independent of known risk factors. This finding suggests that accelerated biological aging may contribute to the pathogenesis of arterial aging through biological pathways beyond established risk factors. The causal impact of telomere shortening on arterial aging warrants further investigation in future studies.

## METHODS

### Study population

The SHFS (2001-ongoing) is a family-based longitudinal study designed to identify genetic, metabolic, and behavioral factors for CVD, diabetes and their risk factors in American Indians in tribes and communities residing in Arizona, North/South Dakota, and Oklahoma. The study enrolled a total of 3,665 tribal members from 94 multigenerational families (14 - 93 years). All living participants were re-examined about 4-5 years later. At each visit, participants underwent a personal interview and physical examination. Information on sociodemographic factors, disease history, medication use, and lifestyle was collected by personal interview using standard questionnaires. A physical examination was conducted and fasting blood samples were collected for laboratory tests, including fasting glucose, lipids, and other biomarkers. The SHFS protocol was approved by the Institutional Reviews Boards from the Indian Health Service and the participating institutions and described elsewhere [40]. In the current study, 874 participants from one community were removed due to withdraw of consent. However, the subsample of 2,791 participants did not differ significantly from the total sample of 3,665 participants in the distribution of risk profiles including demographic and known clinical factors listed in Table 1. After excluding 626 participants with missing data on carotid ultrasound examination or telomeres, a total of 2,165 participants were included in the final data analysis.

### Leukocyte telomere length measurement

Methods for LTL measurement in the SHFS have been described elsewhere [6]. Briefly, genomic DNA from peripheral blood was isolated according to standard methods. LTL of the derived DNA was measured using quantitative PCR through a high-throughput telomere length assay system at Dr. Blackburn's lab at the University of California, San Francisco. LTL was estimated by the ratio of telomeric product/single copy gene (T/S) obtained using quantitative PCR according to protocols reported previously [41]. T/S ratio was calculated by taking the difference between the mean of two T values and two S values attained for each of the three replicates. These three T/S ratios were averaged, and standard deviation and percent of coefficient variation (%CV, standard deviation/mean) were calculated. The T/S ratios were normalized to the mean of all samples and reported. For quality control, we included seven control DNA samples from various cancer cell lines in each assay plate. These control samples allowed us to create standard curves, which were then integrated into a composite standard curve, used for T and S concentration calculations. In addition, about 20% of the samples selected randomly were measured twice. Intra- and inter-assay %CV was 4.6% and 6.9%, respectively.

### Carotid ultrasonography

Participants received carotid ultrasonography examina-tion using Acuson Sequoia machines equipped with 7 MHz vascular probes on the day of the study visit according to a standardized protocol as previously described [13]. Arterial diameter data were collected on the bilateral common carotid artery during carotid ultrasonography examination. In brief, after the participants had rested in a supine position for more than 20 minutes, two-dimensionally-guided M-mode tracings of both the right and left distal common carotid artery approximately 1cm proximal to the bulb were obtained to measure lumen diameter at end-diastole (D_d_) and peak-systole (D_s_). These data were digitized by an analog-to-digital converter, and then sent to the Reading Center for further processing. The arterial diameter data were estimated as the average over several cardiac cycles. The reading of arterial diameter data was performed by a highly experienced cardiologist who was blinded to the clinical characteristics of the participants.

### Aortic pressure measurement

Aortic systolic (P_s_) and diastolic (P_d_) pressures were obtained from applanation tonometry performed immediately after carotid ultrasonography with the position of the subject and ambient environment unchanged. The methods for applanation tonometry in the Strong Heat Study was reported elsewhere [13]. Briefly, applanation tonometry was performed using a solid state high-fidelity external Millar transducer. Aortic pressures were calculated by the use of the SphygmoCor device using a generalized transfer function (AtCor Medical, Sydney, Australia) and calibration using the brachial mean and diastolic pressures obtained immediately before the procedure. Orientation and pressure applied to the transducer were adjusted to optimize applanation of the artery between the transducer and the underlying tissue.

### Arterial stiffness estimate

Arterial stiffness was estimated from pressure-diameter relations of the left and right common carotid arteries. The arterial stiffness index β was calculated according to the formula: ln(P_s_/P_d_)/([D_s_-D_d_]/D_d_) [42], where P_s_ and P_d_ denote aortic systolic and diastolic pressures, respectively, and D_s_ and D_d_ denote carotid systole and diastole diameters, respectively. The higher SI value of bilateral common carotid arteries was used to assess arterial stiffness. This approach could reflect more accurately the severity of arterial stiffness than one-side artery and has been used in previous studies [43].

### Risk factors assessments

Sociodemographics (age, sex, education level) was collected by standard questionnaires. Cigarette smoking was classified as current smoking, past smoking and never smoking. Current smoking was defined as having smoked at least 100 cigarettes in the entire life, having smoked cigarettes regularly, and smoking currently. Past smoking was defined as having smoked at least 100 cigarettes in the entire life, having smoked cigarettes regularly in the past, but not smoking currently. Never smoking was defined as never smoked or having smoked fewer than 100 cigarettes in the entire life. Alcohol consumption was classified as current drinkers, former drinkers and never drinkers as previously described [44]. Current drinkers were those who had consumed any alcohol during the past year, former drinkers had stopped consuming alcohol for ≥ 12 months, and never drinkers were those who reported never drinking alcohol in their life time. Each participant was asked to wear a pedometer for seven consecutive days and to record the number of the steps taken daily in an activity diary. Physical activity was assessed by the mean number of steps per day calculated by averaging the total number of steps recorded in 7 consecutive days. Body weight (kg) and height (cm) were measured when participants wore light clothes and no shoes by trained staff. Body mass index (BMI) was calculated by dividing weight in kilograms by the square of height in meters (kg/m^2^). Waist circumference was measured at the level of the umbilicus while the participant was in a supine position. Blood pressures were measured three times by trained staff using a standard mercury sphygmomanometer after the participants had been resting for at least 5 minutes and the mean of the last two measurements was used in statistical analysis. Fasting glucose, serum creatinine, and blood lipids, including total cholesterol, triglycerides, low density lipoprotein cholesterol (LDL-C) and high density lipoprotein cholesterol (HDL-C), were measured by standard laboratory methods [45]. Diabetes was defined as fasting glucose ≥ 126 mg/dL or receiving medications on diabetes [46]. Estimated glomerular filtration rate (eGFR) was estimated based on serum creatinine by using the Modification of Diet in Renal Disease Study equation [47]. Chronic kidney disease was defined as eGFR < 60 mL/min/1.73 m^2^ [48]. Serum high-sensitivity C-reactive protein (hsCRP) was measured by an enzyme-linked immunosorbent assay (ELISA) developed in-house using purified CRP and anti-CRP antibodies from Calbiochem at the University of Vermont laboratory [49]. Medications on hyper-tension, diabetes, and hypercholesterolemia were obtained by medical records review and standard questionnaires.

### Statistical analysis

Baseline characteristics of study participants were presented according to LTL quartiles. Arterial stiffness index β was log-transformed (i.e., log-SI) to improve normality. To examine the association between LTL and arterial stiffness, we constructed multivariate generalized estimating equation (GEE) model, in which log-SI was the dependent variable and LTL was the independent variable, adjusting for sociodemographics (age, sex, education level), study site, metabolic factors (fasting glucose, LDL-C, HDL-C, systolic blood pressure, and eGFR), lifestyle (BMI, smoking, drinking, and physical activity), and prevalent CVD (y/n). GEE model was used here to account for the family relatedness among participants. To examine whether and to what extent inflammation could potentially confound the association between LTL and arterial stiffness, we further adjusted for hsCRP in the GEE model described above. As prior studies reported that arterial stiffness was elevated in patients with prevalent CVD, diabetes, and chronic kidney disease [12, 13, 30, 31], we conducted additional analyses by excluding participants with prevalent CVD, diabetes, or chronic kidney disease to examine whether these chronic conditions influence the relationship between LTL and arterial stiffness. A two-tailed *P*-value < 0.05 was considered statistically significant. All statistical analyses were conducted using SAS statistical software (version 9.4, Cary, North Carolina).

### Sensitivity analysis

As previous studies reported that some medications on hypertension, [27] diabetes,[28] and hypercholestero-lemia [29] may have favorable effects on arterial stiffness, we thus performed sensitivity analysis by additionally adjusting for the use of medications (y/n) on hypertension, diabetes, and hypercholesterolemia to examine whether and how these medications will affect our results.

## SUPPLEMENTAL DATA TABLE


