# Supplementary material for: Impact of biological aging on arterial aging in American Indians: findings from the Strong Heart Family Study
Source: Aging (Albany NY). 2016 Aug 11;8(8):1583–91. doi: 10.18632/aging.101013 (PMC5032684; doi:10.18632/aging.101013)
Supplement: Supplementary file 1 [file aging-08-1583-s001.pdf]

SUPPLEMENTARY DATA

Supplementary Table S1. Multivariate-adjusted association between LTL and log-transformed stiffness index β in American Indians after further adjusting for medication use

Subgroups	No. of participants	Multivariate-adjusted*		Additionally adjusted for hsCRP†	
		β (SE)	*P*-value	β (SE)	*P*-value
All participants	2165	-0.069 (0.026)	0.007	-0.070 (0.025)	0.005
No CVD	2062	-0.067 (0.028)	0.015	-0.066 (0.027)	0.013
No diabetes	1778	-0.072 (0.028)	0.011	-0.075 (0.029)	0.009
No chronic kidney disease	2033	-0.088 (0.028)	0.001	-0.089 (0.027)	0.001

LTL: leukocyte telomere length; hsCRP: high-sensitivity C-reactive protein.
*Adjusting for sociodemographics (age, sex, education level), study site, metabolic factors (systolic blood pressure, fasting glucose, low- and high- density lipoprotein cholesterol, estimated glomerular filtration rate), lifestyle factors (body mass index, current smoking, current drinking, physical activity), prevalent CVD, and medication use (against hypertension, diabetes, hypercholesterolemia).
†Further adjusting for high-sensitivity C-reactive protein.
